# Temporal Changes in Forest Contexts at Multiple Extents: Three Decades of Fragmentation in the Gran Chaco (1979-2010), Central Argentina

**DOI:** 10.1371/journal.pone.0142855

**Published:** 2015-12-02

**Authors:** Ludovico Frate, Alicia T. R. Acosta, Marcelo Cabido, Laura Hoyos, Maria Laura Carranza

**Affiliations:** 1 Envix-Lab, Dipartimento di Bioscienze e Territorio, Università degli Studi del Molise, c.da Fonte Lappone, 86090, Pesche (IS), Italy; 2 Istituto di Biologia Agro-Ambientale e Forestale, CNR/IBAF, Monterotondo, Roma, Italy; 3 Dipartimento di Scienze, Università degli Studi di Roma Tre, V.le Marconi 446, 00146, Roma, Italy; 4 Instituto Multidisciplinario de Biología Vegetal (UNC-CONICET) and Facultad de Ciencias Exactas, Físicas y Naturales, Universidad Nacional de Córdoba, Av. Vélez Sarsfield 299, C.C. 495, 5000, Córdoba, Argentina; Technion -- Israel Institute of Technology, ISRAEL

## Abstract

The context in which a forest exists strongly influences its function and sustainability. Unveiling the multi-scale nature of forest fragmentation context is crucial to understand how human activities affect the spatial patterns of forests across a range of scales. However, this issue remains almost unexplored in subtropical ecosystems. In this study, we analyzed temporal changes (1979–2010) in forest contexts in the Argentinean dry Chaco at multiple extents. We classified forests over the last three decades based on forest context amount (*P*_*f*_) and structural connectivity (*P*_*ff*_), which were measured using a moving window approach fixed at eight different extents (from local, ~ 6 ha, to regional, ~ 8300 ha). Specific multi-scale forest context profiles (for the years 1979 and 2010) were defined by projecting *P*_*f*_ vs. *P*_*ff*_ mean values and were compared across spatial extents. The distributions of *P*_*f*_ across scales were described by scalograms and their shapes over time were compared. The amount of agricultural land and rangelands across the scales were also analyzed. The dry Chaco has undergone an intensive process of fragmentation, resulting in a shift from landscapes dominated by forests with gaps of rangelands to landscapes where small forest patches are embedded in agricultural lands. Multi-scale fragmentation analysis depicted landscapes in which local exploitation, which perforates forest cover, occurs alongside extensive forest clearings, reducing forests to small and isolated patches surrounded by agricultural lands. In addition, the temporal diminution of *P*_*f*_’s variability along with the increment of the mean slope of the *P*_*f*_ ‘s scalograms, indicate a simplification of the spatial pattern of forest over time. The observed changes have most likely been the result of the interplay between human activities and environmental constraints, which have shaped the spatial patterns of forests across scales. Based on our results, strategies for the conservation and sustainable management of the dry Chaco should take into account both the context of each habitat location and the scales over which a forest pattern might be preserved, altered or restored.

## Introduction

The extent of anthropogenic fragmentation of the natural forests of many tropical and subtropical ecosystems is one of the most severe causes of biodiversity loss worldwide [1]. Typically, a large amount of habitat is progressively reduced into small fragments that are nested within a very different and inhospitable matrix, thereby constituting a serious threat for many species because their populations become smaller and isolated [2] and because they are more susceptible to the edge effect [3] and to the invasion of alien species [4]. Considering suitable habitat patches embedded in an ecologically neutral or inhospitable matrix may represent an over-simplification because very few species have a binary response to habitat [5]; moreover, habitat quality may be strongly affected by the nature of a surrounding landscape (context).

Unveiling forest fragmentation context is crucial for formulating effective measures of conservation and for identifying the specific levels of intervention that are needed to mitigate the negative effects of forest fragmentation on biodiversity [6]. For example, fragmentation reduces the extent of interior forests, which, located in forest-dominated neighborhoods [7], provide natural habitats for true forest species [8–10]. Moreover, fragmentation promotes the incremental creation of forest edges either through the creation of dispersed holes (perforated pattern) or through the progressive, wave-like loss of habitat that begins at one edge of a landscape and then moves progressively across it (edge pattern) [11]. Thus, fragmentation promotes the expansion of edge habitats, which are characterized by specific environmental and biological conditions (microclimate, vegetation, etc.) that differ from those of the interior forest [3, 12]. In severe stages of fragmentation, extensive forests are divided into many small patches where interior forest specialist species tend to disappear and are replaced by edge and generalist species [13, 14]. Yet, fragmentation promotes a juxtaposition of forest and other semi-natural and artificial cover types, which can have a powerful effect on habitat quality that in turn affects species survival probability and reproduction success [6].

When considering forest fragmentation context, scale becomes an important issue because forest context depends on the definition of how much of its surrounding landscape is included in the analysis [7]. Furthermore, habitat fragmentation is a scale-dependent process [15] that is often linked with how and where land-use policies and management strategies interact with the environment [16]. Indeed, a highly fragmented habitat on one scale may be comparatively unfragmented on a much coarser (or finer) scale [5]. In addition, how organisms perceive and experience habitat fragmentation (e.g., dispersal strategies, habitat quality, habitat suitability, etc.) is also scale-dependent [17, 18]. In this regard, single-scale studies are usually of little use, except outside the very specific frames of reference [19], while multi-scale analyses are imperative for obtaining a complete understanding of the spatial patterns and processes of a landscape [16, 20, 21]. Furthermore, the consideration of multiple scales is important for successful biological conservation [22]. In fact, forest management should consider the potential effects of management actions at multiple scales, from single stands to landscapes and regions [23]. An interesting approach to account for forest context fragmentation at different scales was proposed by Riitters et al [20, 15] in which forests are classified into four fragmentation categories based on the amount and pattern of forests present in the surrounding landscape (context). According to Riitters et al [20], a given forest area can be classified as patch, perforated, edge or interior depending on its amount (i.e. the probability of finding other pixels of forest) and structural connectivity (i.e., the conditional probability that a forest pixel is adjacent to another forest pixel) within a specific context, which can be set within a range of window sizes ranging from local to regional (for details, see [20, 15]). This method, based on image convolution, does not require the identification of individual patches. Instead, a fixed-area ‘extent’ is centered over each pixel on a forest map, and the amount and structural connectivity of forest in the window are calculated. This result is then assigned to the forest pixel located at the window center, thus building a new continuous map describing the variation in forest context at each extent. Moreover, an interesting extension of this approach can be implemented to analyze forest fragmentation context in terms of non-forest land-cover types, i.e., the amount of natural, semi-natural and artificial cover types occurring in forest surroundings (context). Forests surrounded by rangelands are expected to experience moderate fragmentation effects on biodiversity respect to forests embedded in artificial or agricultural contexts.

Natural forests in developing tropical and subtropical countries are among the most threatened ecosystems in the world [24]. Major dry forests are especially vulnerable to agricultural clearing and timber extraction, both authorized and illegal, and to forest fires [21–27]. The Gran Chaco, which is among the largest seasonally dry subtropical forests in the world, occurs in Argentina, Paraguay and Bolivia [28–30]. It has been cleared for agriculture and has been replaced by a mosaic of crops, shrublands, secondary forests and primary forest remnants [31, 32]. Although the Gran Chaco is one of the most threatened ecosystems in Latin America [33], its forests are poorly represented in the Argentinean and South American protected area systems [34, 35].

Deforestation processes have been described for different sectors of the Gran Chaco, focusing on forest loss (e.g. [36, 37]) or considering even patch-based parameters (e.g. [31, 32, 38, 39]). However, none of these works have investigated the context of remaining forests or the scale at which forest fragmentation occurs. In this regard, fragmentation context analysis accomplished by the use of a range of window sizes could represent an approach capable of providing both a clear description of forest surroundings and a direct measurement of forest spatial scale [7, 20, 24]. For instance, transitions in forest dominance across scales (interior, perforated, edge, patch) could indicate a landscape where different fragmentation pressures (local and regional) coexist. On the other hand, the presence of patchy forests at all scales might be related to late-stage fragmentation characterized by tiny forest patches dispersed in a non-forest matrix. The dominance of interior forests across scales is likely related to intact and undisturbed landscapes.

In this study, we proposed to extend the fragmentation context analysis of Riitters et al [20] temporally (1979–2010) to examine the effects of landscape change on the spatial pattern of the forests of the Argentinean dry Chaco at multiple scales. We assumed that the influence of fragmentation on forest cover and connectivity over time varies from local to regional scales. In addition, we characterized the forest fragmentation context in terms of non-forest land cover types (rangelands and agricultural lands) across different scales. By identifying the multi-scale behavior of the forest fragmentation context, we develop an in-depth description of the process of fragmentation and provide the basis for the formulation of a scientifically sound hypothesis about the mechanisms driving forest change. Generating an accurate description of the multi-scale nature of forest fragmentation has great potential for conservation planning and for managing landscape fragmentation across scales with predictable benefits for biodiversity.

## Materials and Methods

### Study area

**Fig 1:**
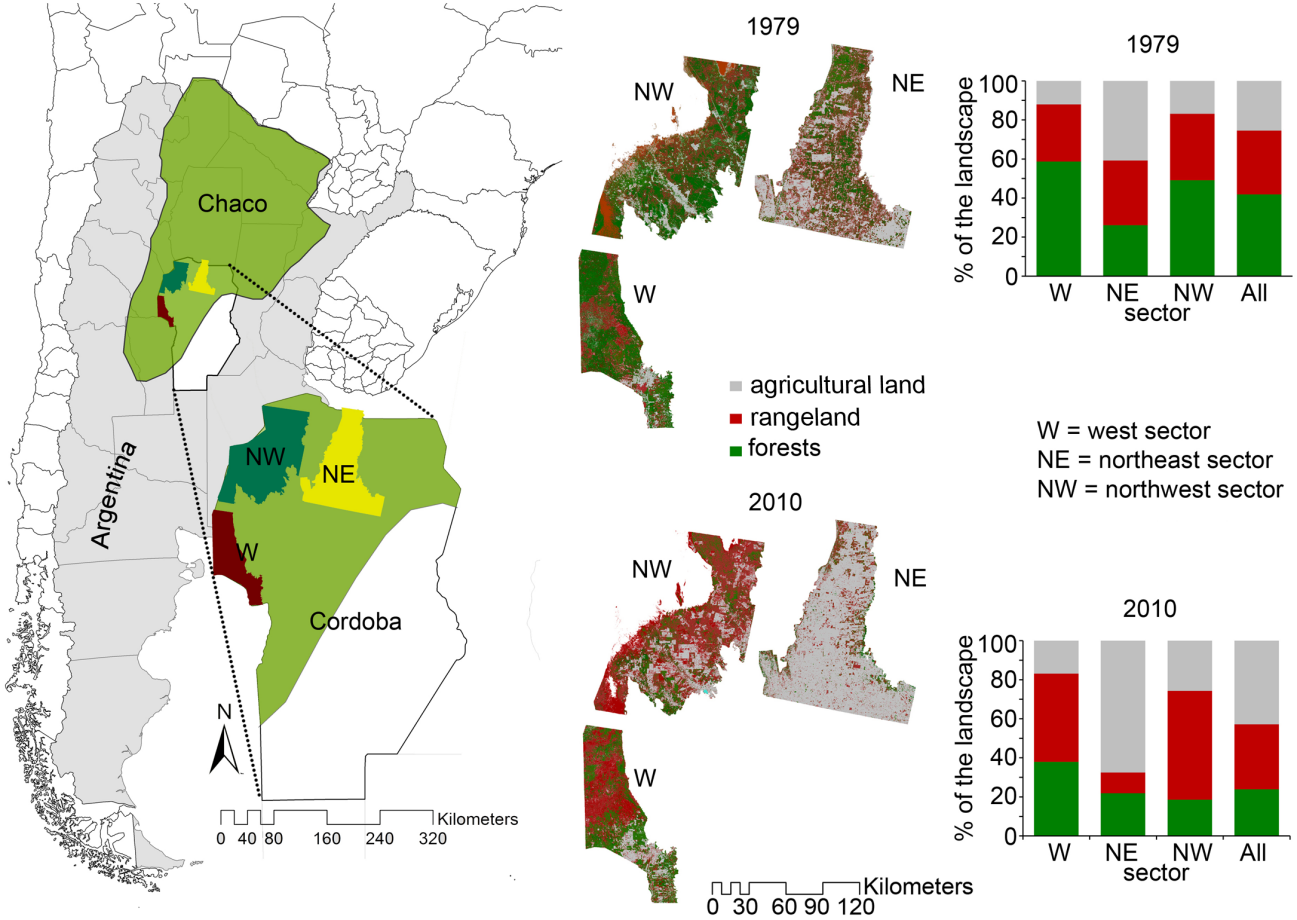
Study area. Location of the Gran Chaco Phytogeographical region, Cordoba Province, and the study sectors in Argentina. Maps of forest, rangelands and agricultural lands along with their relative percent of cover for the years 1979 and 2010 are also reported.

The study area is located at the southern extreme of the dry Chaco, to the northeast and northwest of Cordoba Province in central Argentina (Fig 1). We analyzed a wide area of almost three million hectares in central Argentina arranged into three sectors representative of the Gran Chaco landscape and the main processes that have shaped it over the last decades [32]. The analyzed sectors, designated as "Northeast" (NE), "Northwest" (NW) and "West" (W) have experienced during the last thirty years, different fragmentation due to environmental constraints (mainly climate), technological development and socioeconomic drivers [32, 39]. (Fig 1). The first sector is located east of a mountain range (the eastern semi-arid plain), while the other two occupy the western arid plain, which is west of the same range. The climate is warm temperate to subtropical with a mean annual temperature ranging from 16°C in the NE to 19°C in the NW and W. The mean annual rainfall decreases in the same direction from more than 800 to 500 mm [29]; a pronounced water deficit exists in the northwest and west [40, 29, 32].

The study area is part of the Chaco Phytogeographical Province [41]; its lowlands were formerly dominated by *Aspidosperma quebracho-blanco* (white quebracho) and *Schinopsis lorentzii* (red quebracho) subtropical seasonal forests [36, 40, 42, 43]. Over the last 30 years, the three sectors have experienced a net forest loss of approximately 615,000 ha, which is ~60% of the initial cover (Fig 1) and consistent expansion of agricultural lands and rangelands. The deforestation has been heterogeneously distributed across the three sectors. The northern sectors (NE and NW) have experienced the greatest degree of exploitation, with a net forest loss of 79% and 66%, respectively, in the last 30 years, while this value amounts to 37% in the W sector (Fig 1).

### Land cover and forest cover maps

In this study, the analysis of forest fragmentation was based on land-cover maps derived from Landsat images [32]. We used existing large-scale cover maps of the area for the years 1979 and 2010 [32], which were produced using Landsat satellite images and extensive field data to ensure accuracy [44]. To identify the land-cover units, three Landsat MSS scenes (February 1979; Path/Row: 246/81, 247/81 and 247/82, 79 x 79 m resolution) and three Landsat TM scenes (March 2010; TM, Path/Row: 229/81, 230/81 and 230/82, 30 x 30 m resolution) were classified following the procedure proposed by Zak and Cabido [36] for mapping Chaco vegetation and implemented for the study area by Hoyos et al [32]. All Landsat images, provided by the National Commission for Spatial Activities (CONAE), were acquired during the Southern Hemisphere’s vegetation growing season. The classification procedure can be schematically described in the following steps. First the images were classified using unsupervised classification algorithms. Then, coeval vegetation field data distributed on sampling areas representative of the clusters obtained in the first step where analyzed (Vegetation Database of the Biogeography lab, University of Cordoba, Argentina, which is part of the sPlot database, German Centre for Integrative Biodiversity Research, Halle-Jena-Leipzig). Finally a supervised classification of the images was performed using as training sites a group of sampling areas. Several training sites were used for each class. This classification, based on training sites whose ground characteristics were known, was carried out independently for each scene through a maximum likelihood routine. The validation of the maps was performed using both, field data (Vegetation Database) and visual interpretation of historic ortophotos and “Quickbird” images (Google Earth 2008). A confusion matrix was constructed for the whole area and the Kappa statistic for maps were good (Kappa statistic >0.70). Finally, in order to perform the fragmentation analysis, the spatial resolution of the maps was harmonized to the coarser map (1979), which had a pixel dimension of 79 x 79 m. Five land-cover units were mapped: closed forest, open forest, shrubland, halophytic vegetation and cultural vegetation (cropland plus urban areas). Further details about the construction of the digital maps, confusion matrices and their accuracy assessments can be found in Hoyos et al [32]. A detailed description of cover types is beyond the scope of this paper, however, in brief we could describe: a) closed forests that correspond to lowland, seasonally dry forests, with *Aspidosperma quebracho-blanco* Schlecht. (white quebracho; Apocinaceae) and *Schinopsis lorentzii* (Cris.) Engl. (red quebracho; Anacardiaceae) as dominant trees and a tree canopy of at least 50% cover [32], which according to Sayago [45] comprises the potential vegetation of the area; b) open forests that are characterized by discontinuous tree canopy dominated by (*Prosopis flexuosa* D. C., *Mimozyganthus carinatus* (Griseb.) Burkart and many taxa of the genera *Acacia*) bur the floristic composition is not much different from that of the closed forests; c) shrublands (*Larrea divaricata* Cav.) which act as replacement communities in disturbed sites after pasture or agricultural abandonment and intense fire events, which lead to high bare soil percentages; d) cultural cover types which include agricultural and artificial land areas. For our purposes, we extracted two datasets from the original land cover maps: the first describing forest distribution (corresponding to “Forest land” of the USGS Level I—standard Land Cover Classification System [46]) and the second outlining the presence of other land cover categories in the study area. The first is a binary dataset in which forest class includes closed forests and open forests, and the remaining cover classes are grouped into a single non-forest class (Fig 1). In the second dataset, the non-forest classes were distinguished into two categories referable to the following USGS Level I—standard Land Cover Classification System: rangelands (including semi-natural replacement shrublands) and agricultural land (mainly soybean fields).

### The fragmentation model

**Fig 2:**
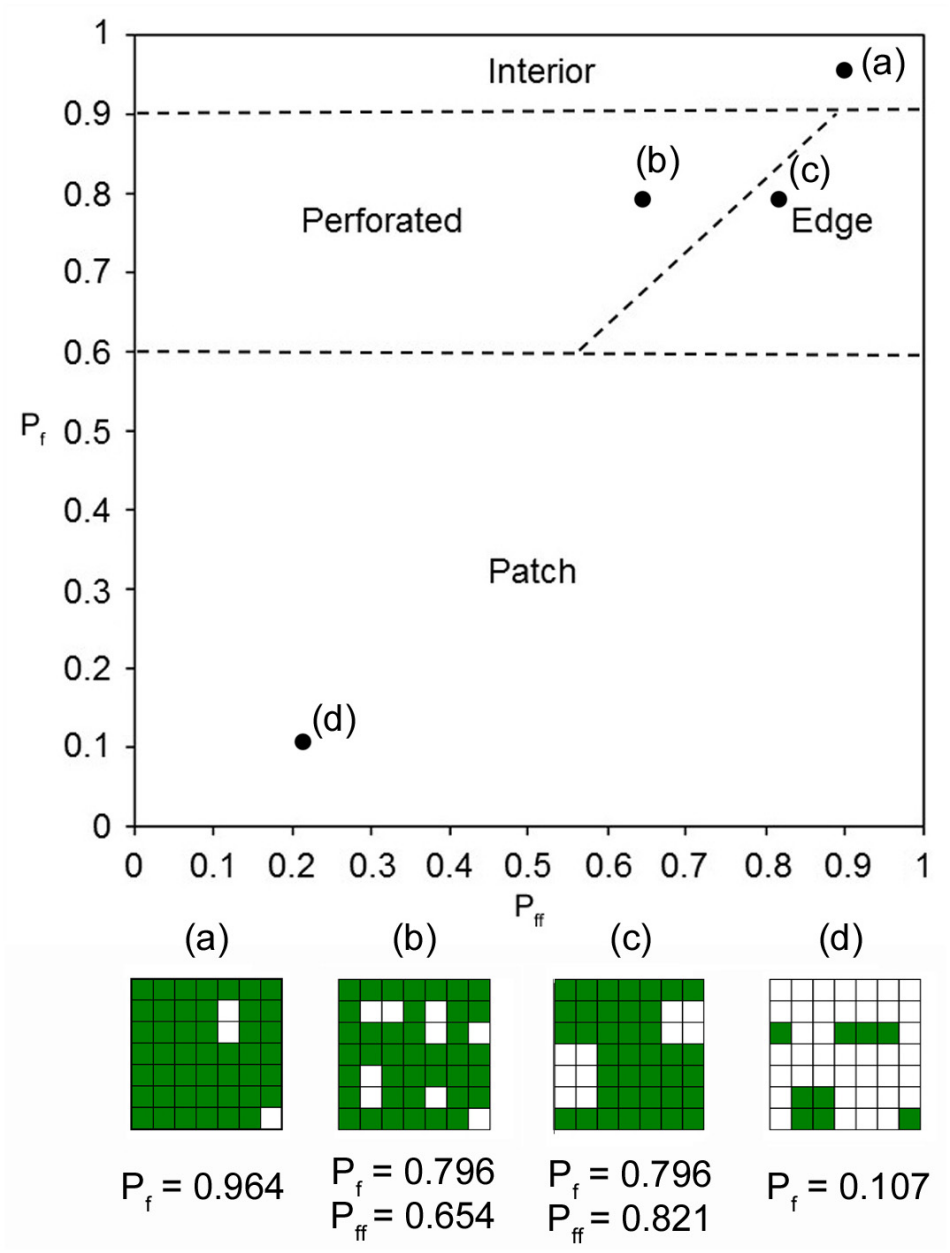
Fragmentation model. Conceptual model used to characterize multi-scale forest fragmentation. *P*_*f*_ and *P*_*ff*_ refer to the amount and the structural connectivity of forest, respectively. Regions of the parameter space corresponding to the “interior”, “perforated”, “edge” and “patch” components are marked. Four simple examples of binary landscapes (a–d) are presented and located on the *P*_*f*_ and *P*_*ff*_ space for different combinations of amount and structural connectivity: (a) undisturbed interior forest landscape, (b) moderately disturbed forests perforated by unforested gaps (i.e., perforated forests), (c) moderately disturbed forests but with clumped disturbed areas (i.e., edge forest), (d) highly fragmented forest (i.e., patch forests). Green: forests; white: non forests.

We described the multi-scale pattern of forest context fragmentation using a moving window device that employed a binary dataset showing pixels of forest and non-forest, and we let composition represent the amount of forested area and configuration denote its spatial arrangement or structural connectivity. The moving window operates by moving a fixed-area window over a map one pixel at a time, calculating the selected metrics within the window and returning that value to the center pixel [17]. The result is a continuous surface that reflects the forested context of each forest pixel. In particular, for each forest pixel, we measured the amount (the probability of finding other pixels of forest- *P*_*f*_) and structural connectivity (the probability that a forest pixel is adjacent to another forest pixel- *P*_*ff*_) within a fixed window size. The simultaneous analysis of *P*_*f*_ and *P*_*ff*_ values in a specific relationship space enables the depiction of a wide range of forest spatial patterns (patch, interior, perforated and edges, Fig 2) (see [20]).

According to Riitters et al [15] recently revisited by Wickham et al [47, 48] when the amount of forest, *P*_*f*_, is above the threshold of 0.6, forests can be considered to dominate the landscape. When the value is >0.9, we refer to the forests as “interior” forests [7]. Interior forests include all forest pixels for which the surrounding landscape is almost completely forested (*P*_*f*_ ~ *P*_*ff*_ ~1). For other “dominated by forest” landscapes (0.6< *P*_*f*_<0.9), we can define a gradient ranging from “perforated” to “edge” forests. In particular, if *P*_*ff*_<*P*_*f*_ then the fragmentation can be said to be “perforated”. In a perforated landscape, a large (relative to landscape size) and non-compact forest cluster alternates with “holes” created by a small amount of non-forested areas (Fig 2). Conversely, when *P*_*ff*_>*P*_*f*_, the fragmentation can be said to be “edge”, such that forests exist in compact clusters next to compact non-forest clusters (Fig 2). When the amount of forest, *P*_*f*_, is below 0.6, forests can be considered as "patch" [15, 47]. Landscapes with low *P*_*f*_ have either a few large forest patches (larger *P*_*ff*_) or many smaller forest patches (smaller *P*_*ff*_), and there is a gradient between the extremes. However, in landscapes characterized by a low cover of forests, overall connectivity is determined less by within-patch forest connectivity and more by between-patch forest connectivity [20].

### Multi-scale context analysis

**Fig 3:**
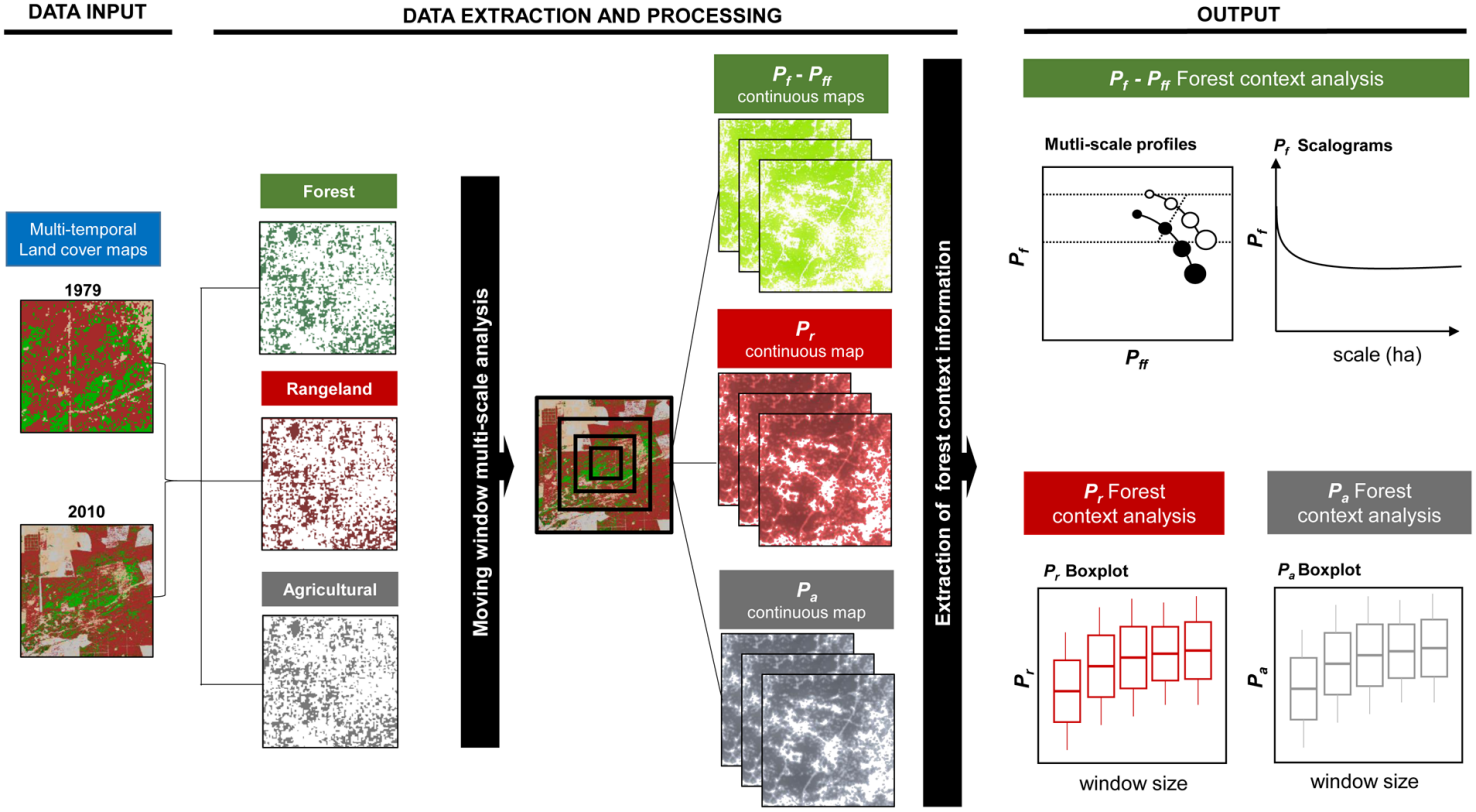
Methodological framework. Flowchart representing different steps of the methodology for detecting temporal changes in forest contexts at multiple extents.

Here, we investigated three aspects of forest context fragmentation over time and across different scales. For each forest location, we describe forest pattern *per se* and the contextual distribution of agricultural lands and rangelands. Thus, context fragmentation analysis was performed on both the forest cover dataset and the land cover dataset (which includes rangelands and agricultural lands). The different steps of the methodology for detecting temporal changes in forest contexts at multiple extents are outlined in Fig 3.

For the forest cover dataset, the amount (*P*_*f*_) and the structural connectivity (*P*_*ff*_) of forests were measured at eight spatial extents using square moving windows of 3 x 3, 5 x 5, 9 x 9, 15 x 15, 25 x 25, 45 x 45, 75 x 75 and 115 x 115 pixels, respectively corresponding to 5.62 ha, 15.60 ha, 50.55 ha, 140.42 ha, 390.06 ha, 1263.80 ha, 3510.56 ha and 8253.72 ha of a real landscape. According to Riitters and Wickham [7], the selected window sizes represent spatial scales of several orders of magnitude to enable tuning up from fine-scale to coarse-scale fragmentation processes. By overlaying the input forest cover map on the continuous *P*_*f*_ and *P*_*ff*_ maps, we extracted context information only for the forest locations.

For each landscape (sectors: NE, NW, E; dates: 1979, 2010), after creating specific maps depicting forest context (see S1 Fig), specific multi-scale forest fragmentation profiles were defined by representing the mean value of *P*_*f*_ ($\bar{P_{ff}}$) versus the mean value of *P*_*ff*_ ($\bar{P_{ff}}$) across the selected range of spatial extents. These mean values denote the average amount of forest and structural connectivity with varying scales. Multi-scale profiles were then compared to describe the fragmentation processes emerging at different scales and to interpret the changes at local, medium and regional scales that have occurred in the dry Chaco over the last 30 years. If a landscape is not fragmented, then the mean amount of forests ($\bar{P_{f}}$) does not decrease with increasing context, and the multiscale profile is nearly horizontal. On the contrary, if a landscape is highly fragmented, the mean amount of forests ($\bar{P_{f}}$) decreases quickly from small to wide extents, and the multi-scale profile is nearly vertical. Of particular interest are the profiles that describe different aspects of forest pattern across scales. For instance, multi-scale profiles that at smaller extents are dominated by interior forest and that drop to patchy forests at medium and large extents describe landscapes where compact forest nuclei (detected at small extents) are embedded within a large, non-forest matrix (detectable only at broader scales). In addition, for each landscape, the statistical distributions of *P*_*f*_ values across the different scales were also represented by scalograms. We performed multi-scale contextual spatial pattern analysis based on the shape of the scalogram [49]. For each scalogram besides $\bar{P_{f}}$, that is habitat average amount of forests across all of the compared scales we analyzed *P*_*f*_ standard deviation (*σ*_*Pf*_), describing the internal variability of *P*_*f*_ at each extent, and the mean slope ($\bar{m}$), which represents the magnitude of the $\bar{P_{f}}$ change across scales. The shape and the variability of each scalogram synthesize forest pattern across scales [50, 49]. As in forest dominated landscapes the scalograms are flat ($\bar{m}$~ 0) with high $\bar{P_{f}}$, and very low *σ*_*Pf*_ values; on fragmented landscapes, scalograms drop from small to medium extents, $\bar{m}$ is high and, *σ*_*Pf*_ decreases at wider scales. On landscapes where local and regional forest exploitation promote the coexistence of large forest clusters with non forest gaps and extensive non forested areas with remnant forest patches, the shape of the scalogram shows a smoother increasing trend; $\bar{P_{f}}$ does not stabilize across scales, $\bar{m}$ is medium and *σ*_*Pf*_ keeps high values even at larger scales.

Then, for the analyzed sectors (NE, NW, E) and dates (1979, 2010), the context of each forest location was also described as the proportion of rangelands and agricultural lands calculated from their surroundings. First, the proportion of rangelands (*P*_*r*_) and agricultural lands (*P*_*a*_) across the selected scales was computed. Second, forest context information was extracted by overlaying the forest cover binary dataset on the continuous density maps describing rangelands and agricultural lands distribution. Third, for each landscape, the statistical distributions of *P*_*r*_ and *P*_*a*_ across scales were represented by box-plots.

## Results

### Forest context fragmentation analysis of the dry Chaco

**Fig 4:**
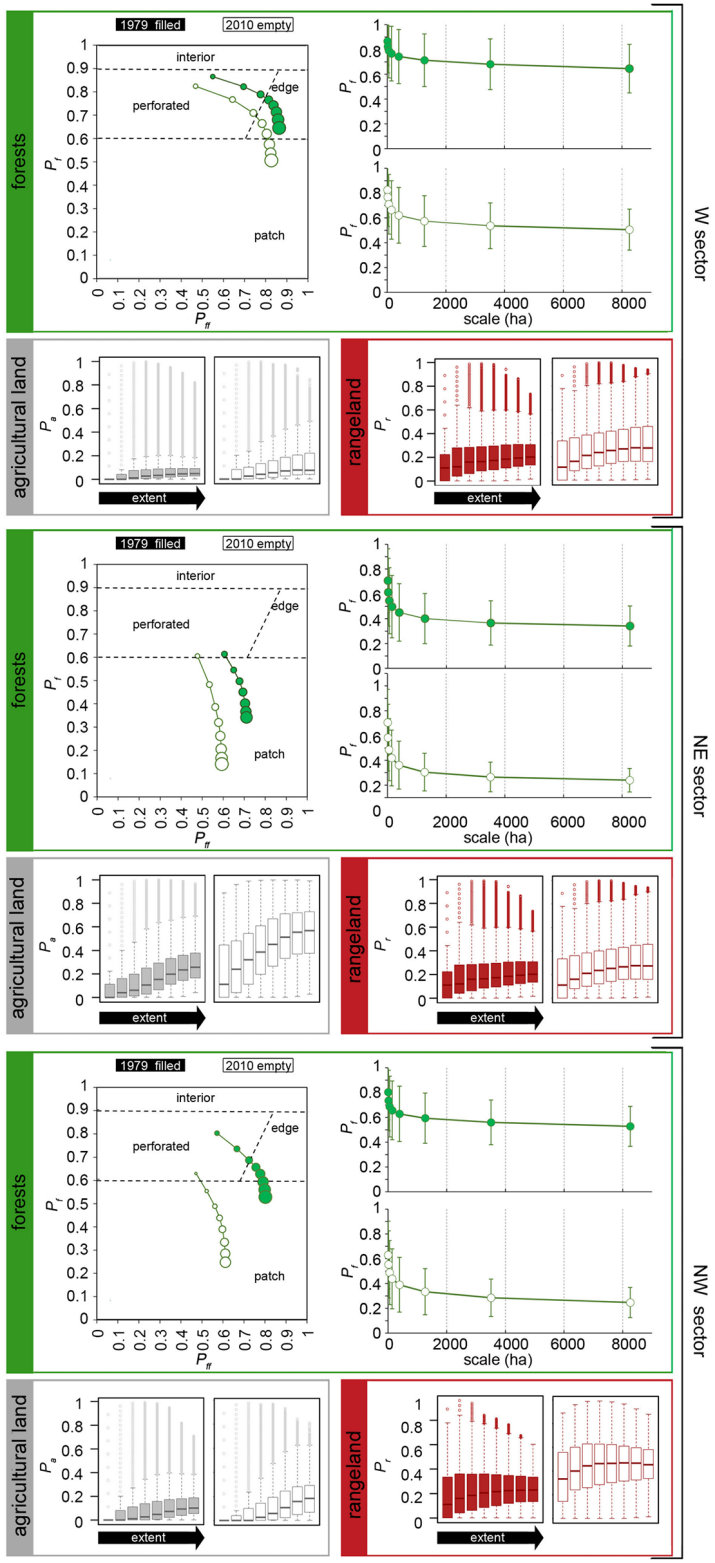
Results of the multi-scale context fragmentation analysis. Multi-scale forest fragmentation profiles of the analyzed sectors (NE, NW, E) and dates (1979, 2010). Symbol dimensions indicate window sizes and increase from small to large extents. Scalograms describing the average amount of forests ($\bar{P_{f}}$), its standard deviations (*σ*_*Pf*_) and box-plots describing the statistical distribution of agricultural lands (*P*_*a*_) and rangelands (*P*_*r*_) on forest context are also represented.

The multi-scale profiles and the scalograms describing Chaco forests and the analysis of agricultural lands and rangelands on their surroundings across scales depict the average characteristics of forest context fragmentation experienced by the analyzed sectors over the last 30 years (Fig 4, Table 1). Forest context fragmentation maps obtained by classifying *P*_*f*_ and *P*_*ff*_ values according with the adopted conceptual model are reported in S1 Fig. In particular, the spatial distribution of interior, perforated, edge and patch forests in each sector, date and scale are mapped.

**Table 1:** Descriptive parameters of the scalograms.

Sector	W				NE				NW			
Year	1979		2010		1979		2010		1979		2010	
Scale (ha)	$\bar{P_{f}}$	*σ*_*Pf*_	$\bar{P_{f}}$	*σ*_*Pf*_	$\bar{P_{f}}$	*σ*_*Pf*_	$\bar{P_{f}}$	*σ*_*Pf*_	$\bar{P_{f}}$	*σ*_*Pf*_	$\bar{P_{f}}$	*σ*_*Pf*_
5.62	0.866	0.194	0.825	0.220	0.708	0.256	0.605	0.267	0.803	0.223	0.630	0.272
15.6	0.823	0.213	0.768	0.237	0.614	0.274	0.483	0.269	0.736	0.244	0.554	0.271
50.55	0.790	0.220	0.710	0.240	0.545	0.267	0.386	0.249	0.687	0.245	0.488	0.258
140.42	0.766	0.220	0.664	0.235	0.497	0.252	0.319	0.225	0.656	0.237	0.438	0.242
390.06	0.743	0.218	0.621	0.224	0.450	0.232	0.262	0.193	0.628	0.224	0.390	0.221
1263.8	0.713	0.212	0.574	0.204	0.401	0.202	0.205	0.152	0.594	0.203	0.334	0.186
3510.56	0.681	0.204	0.537	0.184	0.366	0.179	0.166	0.119	0.560	0.180	0.285	0.151
$\bar{m}$	-0.09		-0.13		-0.2		-0.26		-0.14		-0.16	

*$\bar{P_{f}}$:* mean amount of forests; *σ*_*Pf*_: *P*_*f*_ standard deviation, $\bar{m}$: mean slope of the scalogram.

The multi-scale profiles depict a variety of situations that range from perforated to highly fragmented patchy forests and include a gradient of intermediate behaviors of forest pattern across scales. It is interesting to note that local-scale fragmentation (perforation) is progressively replaced by edge and large-scale fragmentation (patchy pattern) as window size increases (Fig 4).

The multi-scale maps and profiles and the relative scalograms of the W sector (Fig 4 and S1 Fig, Table 1) indicate moderate changes in forest spatial patterns from 1979 to 2010. According to the multi-scale profiles, the landscape was historically dominated by forests at all spatial extents; $\bar{P_{f}}$ values in 1979 ranged from 0.87 at smaller extents to 0.65 at larger scales as connectivity ($\bar{P_{ff}}$) increased from 0.55 to 0.86 (perforated to edge). The analysis of the 2010 profile showed a consistent reduction of *P*_*f*_ and *P*_*ff*_ values across all scales. In 2010, $\bar{P_{f}}$ declined by 0.04 at the smaller scales and by 0.22 at the large scales, and forest loss was five times more evident at larger scales. Unlike that from 1979, the 2010 multi-scale profile shows a shift from a forest-dominated landscape at local scales to a patchy pattern over large scales (45 x 45 window, 1263.80 ha). The shape of the scalograms of the two periods are moderately divergent (Fig 4 and Table 1). The scalogram of the year 1979 is characterized by high values of *P*_*f*_ with *σ*_*Pf*_ that is stable across scales (*σ*_*Pf*_~0.20) and by very low values of mean slope ($\bar{m}$ = -0.09). Instead, in the 2010’s scalogram, *P*_*f*_ and *σ*_*Pf*_ values decrease across scales (*P*_*f*_ decreases from 0.85 to 0.83 and *σ*_*Pf*_ from 0.22 to 0.184) and the mean slope is high ($\bar{m}$ = -0.13). The mean cover of rangelands in forest surroundings and their relative variability (highlighted by the inter-quartile range IQR) increased across scales (0.10 < *P*_*r*_ 1979 > 0.20) and over time with *P*_*r*_ 2010 ~ 0.30 on the wider extents. Instead, agricultural lands cover and variability do not change much across scales and remain quite stable over time (0 < *P*_*a*_1979 > 0.05; and 0 < *P*_*a*_2010> 0.08).

The multi-scale maps and profiles of the NE sector and the relative scalograms (Fig 4 and S1 Fig, Table 1) depict a highly fragmented landscape for both years, where forests are only a minor component of the smallest windows and quickly decrease with increasing extents ($\bar{P_{f}}$ = 0.61, $\bar{P_{ff}}$ = 0.60 for 1979 and $\bar{P_{f}}$ = 0.61, $\bar{P_{ff}}$ = 0.48 for 2010). According to the multi-scale profiles, major changes have occurred in terms of structural connectivity ($\bar{P_{ff}}$), which decreased by approximately 0.15 across all of the analyzed scales. Such diminution indicates a landscape shift towards an even more dispersed forest pattern. The shapes of the scalograms are diferent (Fig 4, Table 1), as the 1979 the values of $\bar{P_{f}}$ and its standard deviation decrease from local to regional scales (*σ*_*Pf*_ from 0.25 to 0.16) and the mean slope of the scalogram is high ($\bar{m}$ = -0.20), in the 2010 the values of $\bar{P_{f}}$ and its standard deviation abruptly decrease across scales (*σ*_*Pf*_ from 0.27 to 0.95) and the mean slope is very high ($\bar{m}$ = -0.26). The mean proportion of rangelands (mainly shrublands) in the 1979 forest context and its variability is stable across scales (0.35 < *P*_*r*_ 1979 > 0.40) and tends to decrease in the more recent data (*P*_*r*_ 2010 ~ 0.20). Conversely, the already high mean cover of agricultural lands in 1979 (0.10 < *P*_*a*_ > 0.20) consistently increased in the year 2010, with values that steeply increased from 0.30 at smaller scales to 0.60 at wider ones (agricultural lands dominates the landscape).

The multi-scale maps and profiles and, the relative scalograms of the NW sector (Fig 4 and S1 Fig, Table 1) show considerable changes in forest pattern from 1979 to 2010. At small and medium spatial extents, the 1979 landscape was dominated by forests (perforated and edge: $\bar{P_{f}}$ from 0.80 to 0.63 and $\bar{P_{ff}}$ from 0.57 to 0.78, respectively), while at large scales (greater than the 45 x 45 = 140.42 ha window) forest cover appeared to be distributed in patches. The multi-scale profiles denote that more recently, the forest pattern has evolved towards a patchy distribution across all scales and, that forest loss was particularly severe at larger scales ($\bar{P_{ff}}$ declined by ~ 0.53 at the broadest scale). This is particularly evident over the 9x9 (50 ha) and 15x15 scales (140 ha), where the majority of forest pixels have a $\bar{P_{f}}$ value smaller than 0.6 (Fig 4). The shape of the scalograms are greatly different. In the 1979 the values of $\bar{P_{f}}$ are intermediate, its standard deviation decreases at larger scales (*σ*_*Pf*_ from 0.22 to 0.16) and the mean slope of the scalogram is high ($\bar{m}$ = -0.14). In the 2010 the values of $\bar{P_{f}}$ and its standard deviation suddenly decrease across scales (*σ*_*Pf*_ from 0.27 to 0.12) and the mean slope of the scalogram is still higher (-0.16). Agricultural lands that historically had little influence on forest context (0 < *P*_*a*_1979 > 0.08) tended to increase over time and reached ~ 0.18 at larger scales. On the other hand, the consistent cover of rangelands that was already present in 1979 (*P*_*r*_1979 ~ 0.20) further increased over time (*P*_*r*_ 2010 ~ 0.40) and its variability tend to decrease across scales.

## Discussion

Over the last 30 years, the dry Chaco has undergone an intensive process of context fragmentation that has affected the spatial patterns of forests at all of the considered scales, from local to regional. Multi-scale context analysis showed that fragmented forests already existed in the 1970s and that the fragmentation process has escalated over the last 30 years. Previous patch-based pattern analyses of deforestation processes at the regional scale have indicated comparable changes in other sectors of the Gran Chaco (e.g., [36, 37, 31]). However, the context approach that was used here provided a clear description of forest surroundings at different extents and enabled the detection of the coexistence of several processes of fragmentation operating at different scales. For instance, using multi-scale context fragmentation profiles, we were able to detect transitions in forest dominance across scales (interior, perforated, edge, patch) and to describe quantitatively a landscape in which local forest exploitation, which perforates forest cover, co-occurs with extensive forest loss, reducing forests to small and isolated patches. The forest context patterns observed across scales and the dynamics of rangelands and agricultural lands in forests’ surroundings are most likely due to the occurrence of different cultural and logging practices [51– 53] as well as the influence of heterogeneous land-use policies enacted at different administrative levels [30].

In particular, the diminution of forest context amount and structural connectivity over time at different scales resulted in a relative increase in forest fragmentation in the W sector. Forest loss occurred at local scales in the 1970s and is still related to forest perforation processes. Such a pattern, along with the very low values of agricultural land and the modest presence of rangelands, is related to the local forest use, cattle grazing activities and traditional agricultural practices that have historically characterized this sector. In the 1979 the landscape is characterized by large clusters of perforated forests that alternates with open areas containing many small and medium forest patches. Such high variability (depicted by the shape of the scalogram) highlight the existence of different landscape processes operating at different scales in the same landscape (scale divergence). In this sector, land exploitation is most likely constrained by the harsh environmental conditions (modest rainfall and clay-rich and salty soils) that have restricted the development of extensive agriculture [54, 29, 24]. On the other hand, the recent patchy forest distribution along with the increment of semi-natural cover types in the forest surroundings observed at larger scales suggest that forest loss has been a local-scale phenomenon that has accumulated to have broad-scale effects [48]. Analogous local deforestation patterns have been observed in the northern Arid Chaco (e.g., [37, 31]), where environmental conditions allow for the diffusion of agriculture parcels, but large forest clearing events by agribusiness are not possible [53]. In the 2010 the existing large clusters of forests diminish and are replaced by medium-sized patches and open areas. Such reduction of landscape variability suggests the smooth onset of a process of landscape simplification. In addition, the increment of rangelands across scales is more likely due to the local production of low-value forest products such as charcoal and fuel wood, as well as the occurrence of fires for cattle grazing activities [40, 55].

Multi-scale context analysis of the NE sector highlighted a dramatic forest change. Having been more than half deforested by 1979, the NE sector was already in an advanced stage of fragmentation. However, it continued to experience significant fragmentation and a consistent increment of agricultural land over the last decades at all spatial scales. The quick decrease of forest cover across scales evidenced by the multi-scale profile along with the reduction of landscape variability across scales (depicted by the scalogram) suggest a critical process of simplification of forest pattern. In this landscape forests are distributed on tiny patches embedded in a wide homogeneous non forested matrix. The observed loss of large forested areas, the reduction of extensive rangelands, and the widespread increment of agricultural lands are most likely related to a combination of good soil conditions [32] and the increase in annual precipitation that was registered over the last half century [54, 29], which together have made the NE sector more suitable for agricultural activities. Indeed, as described by Grau et al [56], the expansion of intensive agriculture driven by global trends in technology has converted large areas of the northwest Chaco and has strongly changed the scale at which forest occurs.

The multi-scale profiles of the NW sector over time illustrate a rapid landscape transition from a perforated to a patchy pattern. In 1979, forests were spatially extensive and alternates with open areas and many small and medium forest patches. Landscape variability is high across all scales denoting the coexistence of landscape processes operating at different scales. For instance, forest perforation processes occurring at local scales and edge fragmentation emerging at wider scales were most likely related to traditional forest harvesting and clearing practices. The temporal changes of forest context profiles and the reduction of landscape variability across scales were triggered by the regional extraction of large forested areas, which were usually replaced by extensive rangelands and subordinated by traditional agriculture. Such changes are most likely related to the historical local exploitation of natural resources, which has intensified over time accompanying the ongoing increase in the mean annual precipitation [32].

Overall, our results suggest that forest fragmentation over time occurs from small to wide extents in the dry Chaco but tends to be more severe at larger scales, such that spatially extensive forests are becoming very rare or are disappearing (especially in the NW and NE sectors). Such changes will likely affect the conditions of the forests themselves [57, 3] and the ability of the forests to supply ecosystem services, such as climate regulation [35], material provisioning [30] and food supply [58]. Furthermore, our findings emphasize the importance of considering the landscape context at multiple scales, which may affect conservation efforts and the sustainable management of dry Chaco ecosystems. Indeed, the nature of the surrounding landscape strongly influences the quality and resilience of forest remnants [59, 60]. For instance, the quick decrease in forest amount and the landscape homogenization at larger spatial extents that has occurred in recent years reveals a worrisome situation. In landscapes near such a critical fragmentation threshold, minor forest loss can dramatically affect the number of patches, the size of the largest patch and the connectivity and quality of the forest remnants [15, 61, 60]. As such effects could be relevant to forests that, as in the NW sector, are surrounded by rangelands dominated by substitution communities, fragmentation effects could crucially affect forests where, as in the NE sector, remaining small forest patches are embedded in agricultural contexts.

Finally, we should note that even though the multi-scale context analysis proposed here offers useful insights to address the influence of spatial extent on forest fragmentation over time, a few sources of uncertainty emerge. For instance the adopted wall to wall forest fragmentation approach partially describe the within landscape variability and forest fragmentation must be interpreted accounting of forest parameters variability (e.g. standard deviation). Furthermore, forest class thresholds are based on percolation theory, which does not strictly apply to real landscapes [62]. Consequently, the interpretation of forest fragmentation near the fragmentation thresholds must be performed with caution and must also include contextual information about other semi-natural and artificial cover types. Nonetheless, the approach that was adopted here accounts for the multi-scalar nature of forest fragmentation and for the quality of the contextual non-forest areas, offering complementary information to the more traditional patch-based or mathematical morphology theory-based approaches.

## Conclusions and Conservation Implications

This multi-scale context analysis of dry Chaco forests depicted an intensive process of fragmentation, resulting in a shift from landscapes originally dominated by forests with gaps of rangelands to landscapes where small forest patches are embedded in agricultural and/or semi-natural cover types. Such changes could have serious consequences for the local biodiversity. As forests of large extent (dominant forests across scales) are necessary to maintain rare and specialist native species [63], the transition towards a non-forested matrix could promote local extinctions, and patchiness at all scales could have serious consequences for the quality of forest remnants. Small patches embedded in an agricultural matrix are particularly susceptible to the influence of edges [64, 65], which can negatively affect the plant diversity of forests [63]. Furthermore, artificial edges may favor the invasion of exotic plant species [64, 4], alter plant reproduction and impair the regeneration of natural flora [66] and fauna [67].

This multi-scale context analysis offers a scientifically sound basis for the conservation and sustainable management of Chaco forests, which still requires the adoption of adequate policies and interventions that account for both the natural forests *per se* and the quality of the forests’ surroundings (context) at different scales. Good examples of multi-scale analysis have been successfully demonstrated for the biological conservation of birds [68] and amphibians [69]. However multi-scale context studies are urgently needed for most of Latin America, where such information is lacking because neither forest context nor multi-scale landscape pattern have been analyzed, and the sustainable management of forest surroundings is not perceived as a conservation tool [35]. Based on our results, any conservation measure should take the context of each habitat location and the scales over which a forest pattern might be preserved, altered or restored into account to achieve specific goals. One strategy for preserving habitat quality and "true forest species" is to preserve the dominant conditions over the range of scales. A different conservation strategy could focus on landscapes that exhibit patchy forest patterns. The conservation value of forest patches may be improved through the management of the surrounding landscape, i.e., by increasing the amount of small-scale interior forests and by improving the quality of forest surroundings. The presence of semi-natural cover types in the forest surroundings could facilitate species dispersal and mitigate the negative effects of anthropic fragmentation on the remaining patches.

This multi-scale context analysis of the dry Chaco has provided evidence for how fragmentation has affected the patterns of the forests at all of the spatial extents considered, altering the scale at which forests occur and reducing the complexity of the landscape mosaic. The observed changes are most likely due to the interplay between human activities and environmental characteristics, which has shaped the spatial patterns of the forests across a range of scales (from local to regional). Indeed, decisions related to forest conversion are typically made at local scales in the Gran Chaco [30], as the expansion of industrial agriculture and large-scale forest clearing events are mainly triggered by trends in regional and global markets [56]. If decisions about landscape management and forest conversion continue to ignore the multi-scale context of forest fragments, huge impacts to the ecological functioning of the Chaco forests would inevitably occur over a range of spatial scales.

## Supporting Information

S1 Fig Forest context fragmentation maps obtained by classifying *P*_*f*_ and *P*_*ff*_ continuous values according with the adopted conceptual model. The spatial distribution of interior, perforated, edge and patch forests in each sector, date and scale are reported.
(JPG)
