# Supplementary figures and images for: Temporal Changes in Forest Contexts at Multiple Extents: Three Decades of Fragmentation in the Gran Chaco (1979-2010), Central Argentina
Source: PLoS One. 2015 Dec 2;10(12):e0142855. doi: 10.1371/journal.pone.0142855 (PMC4667992; doi:10.1371/journal.pone.0142855)

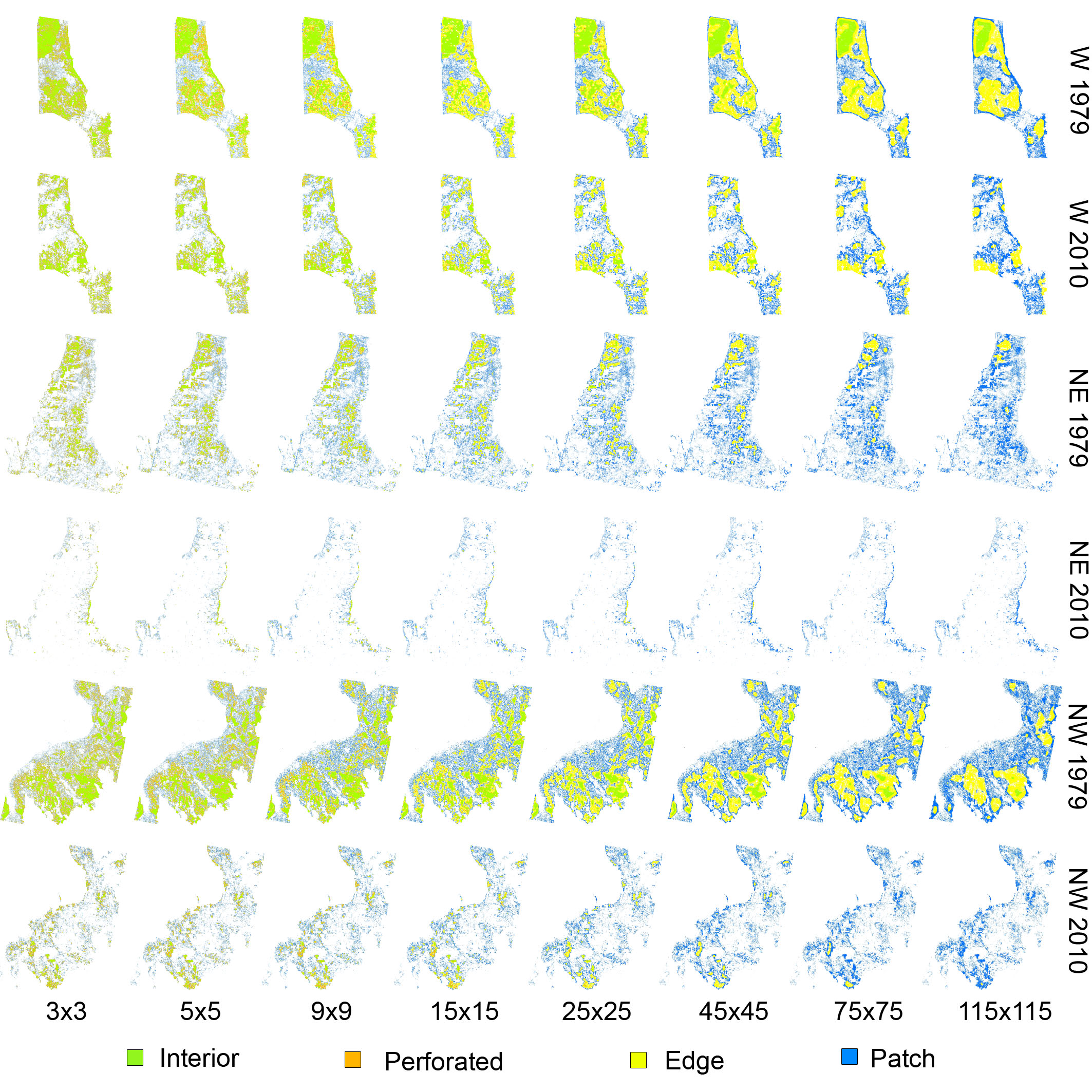

Supplement: S1 Fig — The spatial distribution of interior, perforated, edge and patch forests in each sector, date and scale are reported. (JPG) [file pone.0142855.s001.jpg]
